# Removal of spermatozoa with externalized phosphatidylserine from sperm preparation in human assisted medical procreation: effects on viability, motility and mitochondrial membrane potential

**DOI:** 10.1186/1477-7827-7-1

**Published:** 2009-01-08

**Authors:** Corinne de Vantéry Arrighi, Hervé Lucas, Didier Chardonnens, Ariane de Agostini

**Affiliations:** 1 Unit of Reproductive Medicine, Department of Obstetrics and Gynaecology, Geneva University Hospitals and University of Geneva, 30, bd de la Cluse, 1211 Geneva 14, Switzerland; 2 AB-Biology, AMP74 Center, Hospital Center of Annemasse-Bonneville, France; 3 Reproductive Medecine Center Medixy, La Tour Hospital, Geneva, Switzerland

## Abstract

**Background:**

Externalization of phosphatidylserine (EPS) occurs in apoptotic-like spermatozoa and could be used to remove them from sperm preparations to enhance sperm quality for assisted medical procreation. We first characterized EPS in sperms from infertile patients in terms of frequency of EPS spermatozoa as well as localization of phosphatidylserine (PS) on spermatozoa. Subsequently, we determined the impact of depleting EPS spermatozoa on sperm quality.

**Methods:**

EPS were visualized by fluorescently-labeled annexin V binding assay. Double staining with annexin V and Hoechst differentiates apoptotic from necrotic spermatozoa. We used magnetic-activated cell sorting using annexin V-conjugated microbeads (MACS-ANMB) technique to remove EPS spermatozoa from sperm prepared by density gradient centrifugation (DGC). The impact of this technique on sperm quality was evaluated by measuring progressive motility, viability, and the integrity of the mitochondrial membrane potential (MMP) by Rhodamine 123.

**Results:**

Mean percentages of EPS spermatozoa were 14% in DGC sperm. Four subpopulations of spermatozoa were identified: 70% alive, 3% early apoptotic, 16% necrotic and 11% late apoptotic or necrotic. PS were localized on head and/or midpiece or on the whole spermatozoa. MACS efficiently eliminates EPS spermatozoa. MACS combined with DGC allows a mean reduction of 70% in EPS and of 60% in MMP-disrupted spermatozoa with a mean increase of 50% in sperm survival at 24 h.

**Conclusion:**

Human ejaculates contain EPS spermatozoa which can mostly be eliminated by DGC plus MACS resulting in improved sperm long term viability, motility and MMP integrity. EPS may be used as an indicator of sperm quality and removal of EPS spermatozoa may enhance fertility potential in assisted medical procreation.

## Background

Human sperm quality is defined by the classical parameters, concentration, motility and morphology, according to standard World Health Organisation diagnostic semen analysis [1]. Nevertheless, hidden anomalies affecting spermatozoa membranes such as externalization of the phosphatidylserine (EPS), or spermatozoa organelles such as the disruption of mitochondrial membrane potential (MMP), caspases activation and/or DNA fragmentation, are common somatic features of apoptosis. Such features are not routinely detected in ejaculated spermatozoa but they have been proven to have a negative impact on assisted medical procreation (AMP) outcome [2-8]. Indeed, successful assisted reproduction is mainly dependent on the quality of the sperm plasma membrane, requiring normal integrity and function to provide motility, acrosome reaction, and fertilization [9]. Spermatozoa with impaired membrane integrity occur more frequently in infertile men, partly explaining suboptimal results in AMP. However, striking modifications of sperm plasma membrane occur physiologically in ejaculated sperm. During capacitation, there is an AC/cAMP/PKA-dependent lipid remodelling of the sperm plasma membrane due to phospholipids translocation that lead to externalization of phosphatidylserine (PS) and phosphatidylethanolamine, and to an albumin mediated efflux of cholesterol resulting in an increase in membrane fluidity [10-16].

Apoptosis plays a critical role in many normal biological processes such as embryogenesis and tissue homeostasis, as well as in pathological states and diseases. Apoptosis causes disruption of membrane, phospholipid asymmetry and translocation of PS, a signal for specific recognition and removal of apoptotic cells by phagocytosis [17-20]. Annexin V, an anticoagulant protein of 35.8 kDa, binds outwardly exposed PS with high affinity in a calcium-dependent manner [21]. Thus, the combined use of annexin V and of a supravital fluorescent dye, such as Hoechst 33258, allows the simultaneous detection of apoptotic and necrotic cells [22]. Currently, it is known that germinal cells apoptosis is an underlying mechanism of normal spermatogenesis and an altered apoptotic process has been found to be closely associated with abnormal spermatozoa in semen and thus with male infertility [23-26]. Indeed, Maeda et al. [27] inhibited sperm production in mice by annexin V microinjection into seminiferous tubules, showing that the phagocytic clearance of apoptotic spermatogenic cells by Sertoli cells is PS-mediated and required for a normal production of sperm. In contrast, apoptosis in ejaculated sperm is less well understood and remains controversial [28]. Few reports applied the detection of EPS, by annexin V-labeling, for the evaluation of cryopreservation techniques on ram [29,30], boar [31,32], bull [33-35], and human [36-43] spermatozoa. Other human sperm studies attempted to relate EPS, DNA fragmentation and/or apoptotic proteins with sperm quality parameters [6,7,15,44-56]. It comes out from the literature that EPS on ejaculated mature spermatozoa is either the result of a plasma membrane modification because of capacitation and/or acrosome reaction [11,15,54] or the sign of an early apoptotic phenotype [45,57]. Sakkas et al. [2,58] proposed that apoptosis in ejaculated sperm is the result of a spermatogenetic failure, thus an abortive apoptotic process that started before ejaculation [59]. It has also been described that inflammation [60-62] and bacteria can induce EPS in human spermatozoa [63]. Nevertheless, the link between EPS and sperm apoptosis is still controversial, although it is accepted that annexin V binding to spermatozoa characterizes modified sperm plasma membrane. Interesting results on EPS in relationship with infertility problems in AMP are those of collaborators at the Reproductive Research Center of the Cleveland Clinic (USA), and at the Department of Dermatology and Andrology of Leipzig University (Germany) [39,51,64-70] who adapted the technique of magnetic-activated cell sorting using annexin V-conjugated microbeads (MACS-ANMB) to sperm preparations in order to evaluate the impact of EPS on sperm parameters and fertility potential.

In the present study, we have characterized EPS on spermatozoa after DGC sperm preparation and tested its impact on the quality of sperm prepared for AMP, an essential element for successful fertilization and reproductive success. EPS is the main sperm surface apoptotic marker but its role in ejaculated sperm remains ambiguous. We have investigated the effects of removal of EPS-positive spermatozoa on the motility and MMP integrity of prepared spermatozoa in patients consulting for infertility. To this end, we have depleted sperm preparations of EPS spermatozoa by affinity to annexin V. The patient population studied, consulting for infertility, presented subobptimal semen characteristics and could benefit from removal of EPS sperm, using ANMB-MACS from sperm preparations. This study has tested the feasibility of such purification given the limited amounts of viable spermatozoa available in the ejaculates of these patients. These data allow to draw guidelines for the clinical application of this purification in low concentration and/or bad quality sperm.

Firstly, we determined the proportion of EPS-positive spermatozoa in noncapacitated and capacitated spermatozoa, and secondly we precisely localized PS on spermatozoa among sperms of patients consulting our infertility unit. Further, we compared raw sperm with two sperm preparations, DGC and DGC combined with MACS-ANMB, for percentages of EPS-positive spermatozoa, of viability, of progressive motility and of spermatozoa with disrupted MMP. Collectively, these data allowed to evaluate in details the quality of sperm preparations after elimination of EPS-positive spermatozoa by MACS-ANMB in our population of infertile patients in AMP conditions.

## Methods

### Sperm preparation

This study was approved by the ethic committee of the Gynaecology and Obstetrics Department, Geneva University Hospital, Switzerland.

Human sperm, collected following an abstinence of 3–5 days, were obtained from men attending the Unit of Reproductive Medicine (Geneva University Hospital, Switzerland), after signing a written informed consent. Semen (standard) parameters were thus obtained. After liquefaction, ejaculated sperm were prepared using PureSperm^® ^(NidaCon laboratories AB, Gothenburg, Sweden) or SpermFilter^® ^(Cryos, Denmark) DGC (90–45%). The pellet of the 90% fraction was washed in BM1 medium (Synthetic medium for human in vitro cell culture, NMS BIO MEDICAL AG, Suisse), centrifuged for 10 min at 300 g and resuspended in BM1 medium and placed in an incubator at 37°C in a humidified atmosphere of 6% CO_2_, before applying on MACS-ANMB according to the experimental design. Selection of sperm by DGC was conducted in BM1, a capacitating medium. For capacitation experiments, sperm suspensions were further incubated for 3–5 h at 37°C in a humidified atmosphere of 6% CO_2_. For sperm survival analysis, sperm suspensions in BM1 medium were maintained in the incubator at 37°C in a humidified atmosphere of 6% CO_2 _for 24 h.

Sperm parameters were determined in accordance with the World Health Organization guidelines [1]. Sperm concentration was assessed using a Makler^® ^Chamber. Sperm motility was assessed with 10 μl of suspension between slide and cover-slide. Sperm viability and morphology were assessed using eosin-nigrosin and Papanicolaou staining, respectively [1].

### Experimental design

Sperm count (concentration in million spermatozoa per ml), progressive motility, EPS-positive spermatozoa and MMP-disrupted spermatozoa were evaluated in liquefied sperm (raw samples) after sperm preparations by DGC alone or by combining DGC with MACS-ANMB. We also evaluated sperm survival 24 h following DGC combined with MACS. We determined the sperm recovery rate after MACS preparation. We also applied MACS to low amount of spermatozoa and/or bad quality sperm in order to define the limit of application of this technique.

### Detection and evaluation of EPS-positive spermatozoa in DGC sperm preparation

Prepared spermatozoa were adjusted at a concentration of 0.5–1 million/ml and washed with 1× Binding Buffer (BD Biosciences Pharmingen, San Diego, CA). Spermatozoa were then resuspended in 100 μl 1× Binding Buffer containing 5–10 μl fluorescein isothiocyanate (FITC)-labeled annexin V, according to the manufacturer's instructions (BD Biosciences Pharmingen), and 0.5 μg/ml Hoechst 33258 (Sigma-Aldrich, Switzerland) and incubated for 15 min at room temperature in the dark. Spermatozoa suspensions were washed with Binding Buffer and centrifuged at 400 g. Supernatants were removed and pellets resuspended in 10–15 μl of Binding Buffer and deposited on glass slides. Two types of preparations were done, with or without mounting media for fluorescence, Vectashield (Vector Laboratories, Burlingame, CA), before covering the slide with a coverslip. The first preparation used for the localization of annexin V staining on spermatozoa and the second one for the double labeling with annexin V-FITC and vital dye Hoechst allowing the distinction between spermatozoa with or without EPS, and between intact and dead spermatozoa, respectively. Spermatozoa were analysed under a Nikon fluorescent microscope using a dual filter set for FITC and Hoechst. The specificity of binding of annexin V-FITC to human spermatozoa was demonstrated in control experiments by preincubating spermatozoa with excess purified recombinant annexin V (BD Bioscience Pharmingen) before performing the test or by performing the assay in Binding Buffer in the absence of calcium. Both control conditions prevented the binding of annexin V-FITC to PS-positive spermatozoa. The sensitivity of the assay was tested by using decreasing amounts of spermatozoa and of annexin-V-FITC concentrations.

### Depletion of EPS-positive spermatozoa by magnetic-activated cell sorting (MACS) method using annexin V magnetic beads (ANMB)

The sperm suspensions were divided into two fractions by passage through a magnetic field (miniMACS, Miltenyi Biotec GmbH, Germany) based on the binding of paramagnetic annexin V-microbeads to PS present on the surface of spermatozoa [39,50,71]. Briefly, DGC-prepared spermatozoa were incubated with annexin V-conjugated microbeads (Miltenyi Biotec GmbH) for 15 min at room temperature. About 100 μl of annexin V microbeads suspension were used for each 10 million spermatozoa. The spermatozoa/microbeads suspension was loaded on a separation MS column containing iron balls, which was fitted in a miniMACS separator (magnet) attached to a multistand (Miltenyi Biotec GmbH). The fraction with intact membranes that passed through the column was labeled as MACS-ANMB-negative fraction, depleted in PS, whereas the fraction composed of apoptotic or deteriorated PS-positive membranes spermatozoa was retained in the separation column and labeled as MACS-ANMB-positive fraction. After the column was removed from the magnetic field, the retained fraction was eluted using annexin V-binding buffer (Miltenyi BiotecGmbH).

### Evaluation of EPS

Raw semen samples were washed in BM1 medium before annexin V-FITC staining. Samples of 0.5 to 1 million spermatozoa from either washed raw spermatozoa, DGC or DGC combined with MACS preparations were washed with Binding Buffer, centrifuged at 400 g for 5 min and resuspended in 100 μl Binding Buffer containing 5 μg/ml annexin V-FITC (BD Bioscience Pharmingen) for 15 min at room temperature in the dark. At the end of the labeling, 500 μl Binding Buffer were added to the samples and centrifuged at 400 g for 5 min. 5–10 μl of the antifading Vectashield were added to 10–15 μl of the pellets and deposited on glass slides and rapidly observed under a Nikon fluorescence microscope with a 100-fold objective. At least 200 spermatozoa per slide were examined.

### Evaluation of Mitochondrial Membrane Potential (MMP)

Rhodamine 123 (Rh123) is a cell-permeant, cationic, fluorescent dye that is readily sequestered by active mitochondria without inducing cytotoxic effects. Uptake and equilibration of Rh123 is rapid, within a few minutes. Viewed through a fluorescein longpass optical filter, fluorescence of mitochondria stained by Rh123 appears yellow-green or red with a Rhodamine filter. The staining procedure was performed according to Auger et al. [72]. Briefly, samples of 0.5 to 1 million spermatozoa prepared by either DGC or DGC combined with MACS were incubated in BM1 containing 10 μg/ml Rh123 (Molecular Probes, Leiden, The Nederlands) for 10 min at room temperature in the dark; samples were then pelleted by centrifugation at 400 g, resuspended in BM1 and incubated for 30 min in dye-free BM1 to eliminate the dye not specifically bound to mitochondria. Samples were then pelleted and 15–20 μl were deposited on glass slides and rapidly observed under a Nikon fluorescence microscope with a 100-fold objective. At least 200 spermatozoa per slide were examined.

### Statistical analysis

Results were expressed as mean ± SEM. To compare parameters between raw, DGC and DGC combined with MACS paired or non-paired Student's t-test were used. Potential correlations between sperm annexin V-positivity and MMP integrity and between the amount of spermatozoa loaded on MACS and MACS recovery rate were evaluated by determining Pearson's correlation coefficients and linear regression analysis. P values < 0.05 were considered statistically significant. Statistical analysis were performed using Excel on a HP computer.

## Results

### Identification and quantification of EPS-positive spermatozoa in noncapacitated and capacitated human spermatozoa

During sperm capacitation and/or acrosome reaction as well as early in apoptosis there is a loss of the asymmetry of the plasma membrane phospholipid PS. Annexin V binding to spermatozoa indicates the translocation of PS (EPS) from the inner to the outer layer of the plasma membrane. Using annexin V-FITC binding assay coupled to the vital dye Hoechst 33258, we identified four subpopulations of human DGC-prepared noncapacitated spermatozoa (Table 1). with an average mean of: 1) 70% live intact spermatozoa with no EPS (no fluorescence), namely annexin V-negative and Hoechst-negative (A-/H-), 2) 3% live spermatozoa with EPS (green), namely A+/H-, corresponding to early apoptotic spermatozoa, 3) 16% dead spermatozoa with no EPS but positive for Hoechst (blue), namely A-/H+, and 4) 11% spermatozoa with EPS and positive for Hoechst (blue and green), namely A+/H+, corresponding to late apoptotic or necrotic spermatozoa Nevertheless, there is a great variability among sperm varying from 0 to 15% for the A+/H- subpopulation, 1 to 34% for the A+/H+ subpopulation and from 2 to 38% for total annexin V-positive spermatozoa. We found an average of 14% (11% A+/H+ and 3% A+/H-, annexin V-positivity) EPS-positive spermatozoa following DGC preparation of sperm (Table 1). Similar proportions of EPS-positive spermatozoa were found using the mouse monoclonal IgG anti-Phosphatidylserine, clone 1H6 (Upstate, NY, USA) coupled to an anti mouse IgG-FITC (Sigma-Aldrich) (data not shown).

**Table 1:** Identification and quantification of EPS-positive spermatozoa by Annexin V-FITC labeling in DGC-prepared sperm.

Subpopulations of spermatozoa	DGC-prepared sperm Noncapacitated sperm (n = 17)	DGC-prepared sperm Capacitated sperm (n = 17)
Annexin V-/Hoechst - (%A-/H-)	70.2 ± 5.8 (18–96)	66.2 ± 5.3 (25–91)^S^
Annexin V-/Hoechst + (%A-/H+)	15.7 ± 4.1 (1–52)	13.8 ± 3.6 (2–52) ^NS^
Annexin V+/Hoechst - (%A+/H-)	2.9 ± 0.8 (0–15)	3.6 ± 0.6 (1–8) ^NS^
Annexin V+/Hoechst + (%A+/H+)	11.2 ± 2.4 (1–34)	16.4 ± 2.7 (4–39)^S^
Total Annexin V + (%A+) (%A+/H-)+(%A+/H+)	14.2 ± 2.7 (2–38)	20.1 ± 2.8 (5–41) ^S^
Apoptotic index (A+/H-)/total (H-)	4.5 ± 1.2 (0–22)	5.7 ± 0.9 (1–11) ^NS^

values are mean percentages ± SEM (minimum-maximum values).

**NS **= statistically not significant compared to noncapacitated sperm (paired t-test).

**S **= statistically significant compared to noncapacitated sperm with p < 0.05 (paired t-test).

Our results showed that there is a moderate increase in the percentage of total annexin V-positive (A+/H- and A+/H+) spermatozoa, following incubation in capacitating conditions. This increase concerns only the A+/H+ subpopulation since there is not a statistically significant increase in the early apoptotic annexin V-positive and Hoechst-negative (A+/H-) subpopulation of spermatozoa nor in the apoptotic index, (A+/H-)/total H-, following capacitation (Tables 1 and 2). The increase in EPS-positivity during capacitation involves 47% and 18% (fold increase superior to 1.5 and 2, respectively) of the sperm analysed with a mean percentage increase from 14 to 20% (Table 1).

**Table 2:** Localization of PS on spermatozoa labeled by annexin V-FITC in DGC-prepared sperm

PS localization	DGCprepared sperm Noncapacitated Sperm	(n = 29)	DGC-prepared sperm Capacitated Sperm	(n = 29)
Total (%A-) + (%A+)	100		100	
Annexin V- (%A-)	86.7 ± 1.8 (61–98)		83.7 ± 1.8 (55–98)	
Annexin V+ (%A+)	13.3 ± 1.8 (2–39)	100	16.3 ± 1.8 (2–45)^S^	100
Head		14.7 ± 2.2 (0–46)		18.6 ± 2.0 (0–57)^S^
Head and Midpiece		11.5 ± 1.9 (0–38)		15.4 ± 1.8 (0–44)^S^
Midpiece		7.7 ± 1.4 (0–27)		7.8 ± 1.3 (0–22) ^NS^
Tail		4.7 ± 1.2 (0–33)		5.1 ± 1.4 (0–33) ^NS^
Entire spermatozoon		61.4 ± 3.6 (25–100)		53.1 ± 3.5 (18–89)^S^

Values are mean percentages ± SEM (minimum-maximum values).

**NS **= statistically not significant compared to noncapacitated sperm (paired t-test).

**S **= statistically significant compared to noncapacitated sperm with p < 0.05 (paired t-test)

### Localization of PS on human spermatozoa

**Figure 1:**
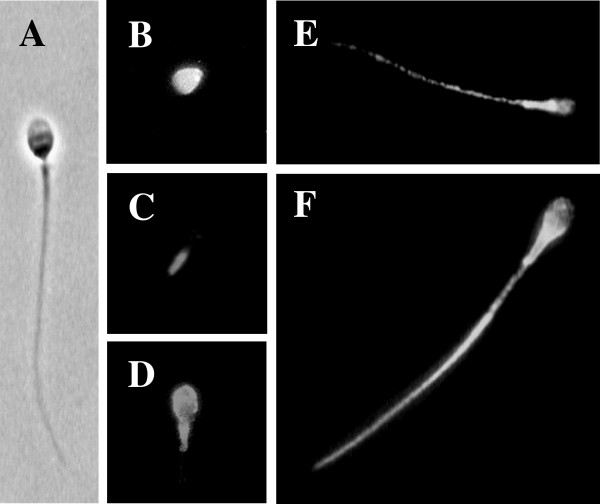
**Localization of PS on human spermatozoa**. PS are visualized by labeling spermatozoa with annexin V-FITC staining. (A) A spermatozoa as seen under bright field microscopy. (B-F) Representative patterns of annexin V-FITC binding sites: head (B), midpiece (C), head plus midpiece (D), tail (E), or entire spermatozoon (F) plasma membrane as seen under fluorescence microscopy. Magnification ×900.

PS was localized on head alone, head plus midpiece, midpiece alone, tail or on the entire spermatozoon (Figure 1 and Table 2). Among the annexin V-positive spermatozoa population (13%), about 15% are labeled on head, 8% on midpiece and 12% on head and midpiece (Table 2). Head PS labeling can concern the apical, basal, equatorial region or entire head, and for midpiece PS labeling, the upper, lower region or whole midpiece. PS localization thus vary among spermatozoa involving either the head and/or midpiece region or the entire spermatozoa. A slight significative increase in annexin V-positivity in the categories head and head plus midpiece was observed under capacitation conditions (Table 2).

### Study design and sperm samples evaluation

**Figure 2:**
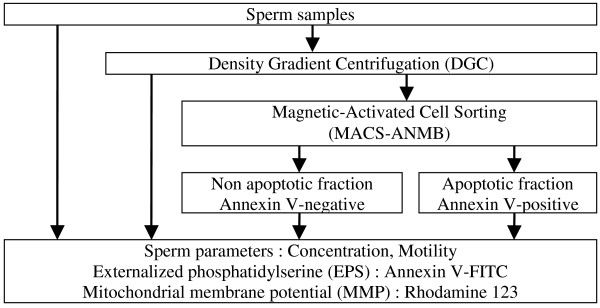
**Diagram of overall experimental design showing the different steps performed in our study**. Number of semen samples analysed for each experimental set: total sperm samples: 44, DGC-EPS: 36, DGC combined with MACS-EPS: 21, DGC-MMP: 31, DGC combined with MACS-MMP: 15, DGC combined with MACS-Motility: 28, DGC combined with MACS-Survival at 24 h: 20 (see also Table 4).

We thus studied 44 sperms of patients consulting our AMP laboratory of the Unit of Reproductive Medicine at our institution. Our study focused on the evaluation of the quality of spermatozoa prepared by MACS-ANMB. Sperm parameters such as concentration and motility, and the apoptotic markers, EPS and MMP, were determined on raw sperm samples, and on sperm samples following DGC or DGC combined with MACS-ANMB preparations (Figure 2). Two fractions were obtained following MACS-ANMB, the non apoptotic, annexin V-negative and the apoptotic, annexin V-positive (Figure 2). Raw sperm parameters are presented in Table 3. Insufficient amount and/or bad quality of some sperm samples led us to partial analysis of our parameters. We thus formed subgroups of sperm according to the parameters analysed, *i.e. *EPS, MMP, progressive motility, and survival at 24 h (Tables 4 and 5). All classical sperm parameters, *i.e*. volume, concentration, total count, motility and morphology are shown in Table 3 and were not statistically different between these subgroups. In raw sperm, EPS-positivity and MMP integrity were inversely correlated (R = -0.37).

**Table 3:** Sperm parameters of patients

Raw sperm Patients	Volume	Concentration (10^6^/ml)	Total Count (10^6^/ejaculate)	Motility (%progressive)	Morphology (%normal forms)
n = 44	3.7 ± 0.2	75.7 ± 7.0	259.1 ± 23.3	49.1 ± 1.9	7.8 ± 0.9

Values are means ± SEM

**Table 4:** EPS, MMP and progressive motility in raw sperm, in DGC and in DGC combined with MACS-ANMB prepared sperm

Criteria, subgroups	Raw sperm	DGC sperm	DGC+MACS sperm Non Apoptotic Fraction	DGC+MACS sperm Apoptotic Fraction
EPS (%A+), n = 36	20.0 ± 2.3 (5–68)	11.8 ± 1.4 (1–32)	-	-
EPS (%A+), n = 21	22.1 ± 3.4 (5–68)	13.1 ± 2.0 (1–32)	6.7 ± 1.2 (1–22)	67.8 ± 4.2 (25–94)
MMP disruption (%MMP-), n = 31	61.7 ± 3.2 (30–95)	44.2 ± 3.7 (16–86)	-	ND
MMP disruption (%MMP-), n = 15	57.9 ± 4.5 (30–91)	37.0 ± 5.0 (16–86)	24.7 ± 5.6 (6–82)	ND
Motility, n = 28 (%progressive)	48.4 ± 2.4 (17–71)	64.4 ± 3.8 (22–91)	67.9 ± 5.4 (0–97)	8.0 ± 2.6 (0–53)

Values are means ± SEM (minimum-maximum values).

All values are statistically significantly different when comparing DGC+MACS, DGC, raw samples (p < 0.05, paired t-test) except for the value of motility 67.9 compared to 64.4.

**Table 5:** Sperm survival at 24 h in DGC and DGC plus MACS-ANMB preparations

n = 20	Raw sperm	DGC sperm	DGC+MACS sperm Non Apoptotic Fraction	DGC sperm Survival at 24 h	DGC+MACS sperm Non Apoptotic Fraction Survival at 24 h
Motility (%progressive)	48.9 ± 3.0 (17–71)	69.3 ± 4.2 (22–91)	75.6 ± 5.2 (20–97)	32.4 ± 6.1 (0–78)	47.5 ± 7.0 (0–88)

Values are means ± SEM (minimum-maximum values).

All values are statistically significantly different (p < 0.05, paired t-test)

### DGC combined with MACS-ANMB was efficient in reducing the sperm population in EPS-positive spermatozoa

The mean percentage of EPS-positive spermatozoa in raw sperm samples was 22% (n = 21), this value fall to 13% following DGC preparation and to 7% after DGC combined with MACS-ANMB (Table 4). DGC sperm preparation, which is the routinely used method of sperm preparation in AMP laboratories, allowed a reduction of about 40% in EPS-positive spermatozoa. This reduction in EPS-positive spermatozoa was similar to that found for the larger sperm samples subgroup, n = 36 (Table 4). The use of the MACS-ANMB technique allowed a further reduction of about 50% of EPS-positive spermatozoa in the non apoptotic annexin V-negative fraction. In other words, we saw a decrease of minus 9% EPS-positive spermatozoa in DGC compared to raw sperms, and a further decrease minus 6.4% EPS-positive spermatozoa in MACS-ANMB-DGC compared to DGC-prepared sperms (Table 4). The sensitivity and specificity of the MACS-ANMB technique for the separation of EPS-positive spermatozoa was evaluated by labeling the unbound annexin V-negative fraction and the bound annexin V-positive fraction from each sperm sample with annexin V-FITC and by visualization under a fluorescent microscope. On average, 67.8% spermatozoa were recognized as EPS-positive in the annexin V-positive fraction and 6.7% were recognized as EPS-positive in the annexin V-negative fraction (Table 4).

### DGC combined with MACS-ANMB had an effect on the quality of spermatozoa in terms of MMP integrity and progressive motility

The mean percentage of MMP-disrupted spermatozoa in raw sperm samples (n = 15) was 58% and this value fall to 37% following DGC preparation and to 25% after DGC combined with MACS-ANMB (Table 4). DGC sperm preparation allowed a reduction of about 30% in MMP-disrupted spermatozoa. A comparable result was obtained for the larger subgroup with 31 sperm samples (Table 4). The use of the MACS-ANMB technique allowed a further reduction of about 30% in MMP-disrupted spermatozoa in the non apoptotic annexin V-negative fraction. In other words, we observed a decrease of minus 21% MMP-disrupted spermatozoa in DGC compared to raw sperms, and a further decrease of minus 12% MMP-disrupted spermatozoa in MACS-ANMB compared to DGC-prepared sperms (Table 4).

DGC sperm preparations greatly enriched the population of progressive motile spermatozoa, categories 3 and 2 according to World Health Organisation [1]. In our experimental conditions, the addition of the MACS-ANMB technique to DGC preparations only slightly improved the sperm motility. In other words, we report an increase of plus 16% (n = 28) and plus 20% (n = 20) spermatozoa with progressive motility in DGC compared to raw sperms, and an increase of plus 3.5% (n = 28) and plus 6% (n = 20) spermatozoa with progressive motility in MACS-ANMB compared to DGC-prepared sperms) (Table 4 and 5).

### Positive effect of DGC combined with MACS-ANMB on sperm survival

Mean progressive motility of raw sperms (n = 20) was increased by 40% by DGC preparation (69% compared to 49%) and further increased by 10% by passage through MACS-ANMB (76% compared to 69%) (Table 5). To further study the increased viability and motility after removal of EPS spermatozoa from prepared sperm, we evaluated the motility 24 h following sperm preparation by DGC or DGC combined with MACS-ANMB (Table 5). The results showed that sperm survival at 24 h expressed in mean percentage of progressive motility was higher in DGC combined with MACS-ANMB preparations than in DGC preparations alone, with approximately 48% against 32% progressive motile spermatozoa, respectively. Sperm survival at 24 h was thus increased by 50% by passage through MACS-ANMB, an increase considerably more pronounced than the 10% increase in progressive motility observed rapidly following the passage through MACS-ANMB (Table 5).

### Sperm recovery rate after MACS-ANMB preparation

The MACS-ANMB technique in our experimental conditions, including infertile sperms of either low motility rates, poor morphology and/or low amount of spermatozoa (0.5 to 10 millions), allowed us to recover a mean of 76% of the quantity (million) of spermatozoa loaded on MACS-ANMB column (Table 6). Nevertheless, the recovery rate of spermatozoa expressed in percentage of spermatozoa charged on the MACS-ANMB column, washing and resuspension in BM1 medium, greatly vary among sperm, from a minimum of 36% to a maximum of 100% recovery when compared with spermatozoa recovered at the end of the preparation after passage through the column. There is a significant positive correlation between the amount of spermatozoa (in million) loaded on MACS and the MACS recovery rate (R = 0.48). The percentage of spermatozoa collected in the MACS-ANMB-negative fraction, corresponding to the non apoptotic fraction, was higher than in the MACS-ANMB-positive fraction, corresponding to the apoptotic fraction, 89% and 11%, respectively (Table 6).

**Table 6:** Sperm recovery rate

Sperm n = 28	Loaded on MACS	Recovered from both fractions	Recovered from the Non Apoptotic Fraction	Recovered from the Apoptotic Fraction
Million	6.74 ± 0.63 (0.5–10)	5.30 ± 0.62 (0.28–10)	4.87 ± 0.1 (0.24–9.95)	0.43 ± 0.1 (0.01–1.5)
%	100%	75.8 ± 3.7*		
%		100%	89.2 ± 2.2*	10.8 ± 2.2*

Values are means ± SEM in million spermatozoa (minimum-maximum values)

## Discussion

Several studies have shown that ejaculated spermatozoa do exhibit changes consistent with apoptosis in particular EPS, decrease in MMP integrity, DNA fragmentation and expression of a number of pro- and anti-apoptotic proteins [73]. Unfortunately, DGC preparation, which is the technique currently used for sperm preparations in AMP laboratories [74], does not eliminate all spermatozoa with apoptotic features. The present study was carried out to quantify EPS-positive spermatozoa and localize PS on spermatozoa in a population of infertile patients attending our *in vitro *Fertilization laboratory and to measure the impact of removing these EPS-positive spermatozoa by MACS-ANMB on sperm quality.

We determined in raw and DGC-prepared sperm from infertile patients the proportion of spermatozoa with compromised survival and fertility potential. In raw sperm, a mean of 20% (min 5%–max 68%) are annexin V-positive (Table 4). Following DGC, about 30% of the spermatozoa are still in late apoptotic or necrotic state and about 14% are annexin V-positive (Table 1).

The moderate increase in annexin-V-positive spermatozoa observed following incubation in capacitating conditions (involving only the A+/H+ subpopulation, Table 1) might be due to the in vitro incubation. Indeed, the number of live sperm drops quickly over time in samples from patients with poor semen quality. We cannot rule out the possibility that the A+/H- is a quick and transitory subpopulation being converted in the A+/H+ subpopulation [49], as also suggested by Sion et al. [43]. Kotwicka et al. [12] detected an increase from 15% to 35% annexin V-positive spermatozoa following incubation in capacitating medium. Martin et al. [54] showed that EPS is mainly related to the acrosome reaction because calcium ionophore A23187 induced a significant increase in the proportion of living EPS-positive spermatozoa from 7 to 48%.

Results from the literature are still controversial. Some authors reported that EPS was related to sperm capacitation process due to a bicarbonate-activated outward translocation of PS [11,15,54]. In contrast, other reports found correlations between EPS and apoptotic events such as DNA fragmentation, caspases activities and/or loss of MMP integrity [6,7,44-47,49,50,52,53]. Taken together, EPS spermatozoa could be survivors of a testis abortive apoptotic process or the result of oxidative stresses initiated during the transit or storage in the male genital tract or be linked to a physiological event in the post-ejaculation period. We could also speculate that spermatozoa might enter an apoptotic-like pathway, giving a physiological role to EPS during capacitation/acrosome reaction, for the acquisition of fertilizing ability, in a natural mechanistic economy. Such mechanism would be dependent on a correct schedule for successful fertilization [75], or otherwise it would be converted in the apoptotic signal for elimination by phagocytic cells. Indeed, it is known that many boar spermatozoa are phagocytized after insemination, thus reducing the risk of eliciting a harmful immune reaction that may interfere with fertilization or embryos implantation [76]. Altogether, regardless of whether EPS represent, an apoptotic marker or a capacitated/acrosome reacted marker, EPS-positive spermatozoa in fresh ejaculates are potentially harmful in AMP being in apoptosis or off schedule for fertilization, respectively.

Concerning the localization of PS on human spermatozoa (Figure 1 and Table 2), we found several categories of annexin V-positive spermatozoa. Our results are similar to those of Shen et al. [49] who also reported annexin V-labeling around head, midpiece and certain parts of the tail. Following incubation in capacitating conditions we found an increase in both head and head plus midpiece categories. Kotwicka et al. [12] also found a change in the localization of PS during capacitation, firstly mainly in the midpiece and then in the acrosomal region of the head. de Vries et al. [15] reported that PS in living spermatozoa was restricted to the apical area of the head plasma membrane, whereas deteriorated spermatozoa presented PS at the midpiece. The various PS localizations on spermatozoa could thus be interpreted as different stages of the dynamic physiological process of capacitation/acrosome reaction or apoptosis versus necrosis status.

Numerous reports linked fertilization failures to apoptotic markers [8,45,77,78]. Infertile patients have higher rates of spermatozoa with EPS, activated caspases and/or loss of MMP integrity [7,45,50]. Therefore MACS-ANMB, which eliminate EPS-positive spermatozoa, is adapted to human sperm in the context of seeking new techniques with better criteria of selection for sperm preparations in AMP [39]. Our study on sperm from infertile patients shows that DGC combined with MACS-ANMB is: 1) applicable to low amount, 43% of sperm tested with less than 5 million spermatozoa, and/or bad quality spermatozoa, 2) efficient in removing EPS spermatozoa and, 3) efficient in enhancing the quality of spermatozoa in term of MMP integrity, progressive motility and most importantly in terms of sperm survival. In our serie of experiments the sensitivity and specificity of MACS-ANMB technique for the separation of EPS-positive spermatozoa (Table 6) are very similar to that reported by Paasch et al. [50,79], except that we labeled spermatozoa by annexin V-FITC and observed them by fluorescence microscope rather than labeling with FITC-conjugated anti-annexin V antibodies followed by FACS analysis. Indeed, in the apoptotic fraction a mean of 67.8% (n = 17 infertile patients, Table 4) of spermatozoa were recognized as EPS-positive compared to 72.2% (n = 30 with15 infertile and 15 fertile donors, [79] and in the non apoptotic fraction a mean of 6.7% (n = 21 infertile patients, Table 4) were recognized as EPS-positive compared to 5.2% [79]. Thus, DGC combined with MACS-ANMB was efficient in removing EPS-positive spermatozoa. MACS-ANMB allows a further decrease of 50% in EPS spermatozoa, with a mean of less than 7% EPS-positive spermatozoa in the non-apoptotic fraction.

We show that combining MACS-ANMB with DGC for sperm preparations had a positive effect on spermatozoa MMP integrity (Table 4). MACS-ANMB allowed a further reduction of 30% in MMP-disrupted spermatozoa, with a mean of 75% of spermatozoa with MPP integrity, compared to DGC alone. Removal of MMP-disrupted spermatozoa is of interest in AMP, since the functionality of mitochondria differentiates human spermatozoa with high and low fertilizing ability [80]. MACS-ANMB combined with DGC also enhanced spermatozoa progressive motility (76% compared to 69% in DGC, +7%) and most outstandingly sperm survival at 24 h (48% compared to 32% in DGC, +16%), however the data showed a great individual variability among sperm from minus 35% to plus 75% (Table 5). In a similar way, the use of the MACS-ANMB technique prior to sperm cryopreservation allowed a significantly higher cryosurvival rate expressed in motility following cryopreservation-thawing [51,70,81]. The better sperm survival at 24 h in DGC plus MACS-ANMB treated sperm suggested that in addition to a purely quantitative decrease of the percentage of EPS apoptotic spermatozoa, their removal might protect healthy spermatozoa from reactive oxygen species production. Indeed, excessive reactive oxygen species, produced either by the presence of immature and abnormal spermatozoa or seminal leukocytes result in altered semen parameters, including viability, motility and morphology [82-90].

We have also undertaken the evaluation of the extent of spermatozoa loss using MACS-ANMB technique. The average number of lost spermatozoa was 24% (n = 28) which is considerably greater than the 15% obtained by Said et al. [69] following DGC plus MACS. This discrepancy may be explained by the use of low amount of spermatozoa from infertile sperm, which reflect the clinical situation in assisted medical procreation.

## Conclusion

In conclusion, our results demonstrate in infertile sperm that MACS-ANMB is a simple, fast, low cost cell sorting system that can be easily adapted in AMP Laboratories to enhance classical sperm parameters and lower sperm apoptotic markers. These results confirm and expand the data from the groups of Cleveland and Leipzig using normal sperm [39,65-70,81,91,92]. Additionnally, these authors reported that MACS-ANMB technique yielded a sperm population with higher normal morphology, reduced caspase-3 activation and DNA fragmentation. The cryosurvival rates and sperm fertilization potential were also improved as measured by higher oocyte penetration capacity in a zona free hamster sperm penetration assay [69,70,91]. Taken together, sperm preparations combining DGC with MACS-ANMB, should significantly enhance the outcome of AMP in terms of fertilization, embryo quality, pregnancies and birth rates. Even though, Varum et al.[93] suggested that elimination of defective spermatozoa using the surface marker annexin V seems unwarranted because annexin V and DNA nicks did not correlated with ubiquitin labeling. Further investigations need to be conducted to assess safety of the magnetic field and the eventual residual microbeads on spermatozoa [50,70].

Implementation of MACS is very promising for the future of ART because MACS has many potential applications in diverse male infertilities due to the possibility of selecting the antibody of the ligand protein used for sperm selection [70]. Very recently, a newly developed solid phase molecular filtration system combining classical glass wool filtration with PS-binding properties of annexin V has been proven to enrich spermatozoa free of apoptosis markers to the same extent as MACS but without the potential inconvenient of an accidental transmission of supermagnetic microbeads into oocytes [94]. Overall, PS might become the prognostic marker of sperm fertility potential easy to use for sorting spermatozoa in ART. In consequence, discarding EPS spermatozoa may optimize AMP outcomes.

## Abbreviations

AMP: assisted medical procreation; DGC: density gradient centrifugation; EPS: externalization of the phosphatidylserine; FITC: fluorescein isothiocyanate; MACS-ANMB: magnetic-activated cell sorting using annexin V-conjugated microbeads; MMP: mitochondrial membrane potential; PS: phosphatidylserine; and Rh123: rhodamine 123.

## Competing interests

The authors declare that they have no competing interests.

## Authors' contributions

CDVA did the work of acquisition of funding, conception, design, and acquisition of data, analysed and interpreted the data, drafted the manuscript, tables and figures, and revised the manuscript. HL did the work of acquisition of funding, contributed to conception and design, analysed and interpreted the data, and revised the manuscript. DC participated to the discussions, and revised the manuscript for content and language. ADA did the work of acquisition of funding, contributed to conception and design, analysed and interpreted the data, and revised the manuscript. All authors read and approved the final manuscript.

## References

[B1] World Health Organization (WHO) (1999). Laboratory manual for the examination of human semen and sperm-cervical mucus interaction.

[B2] Sakkas D, Mariethoz E, St John JC (1999). Abnormal sperm parameters in humans are indicative of an abortive apoptotic mechanism linked to the Fas-mediated pathway. Exp Cell Res.

[B3] Sakkas D, Seli E, Bizzaro D, Tarozzi N, Manicardi GC (2003). Abnormal spermatozoa in the ejaculate: abortive apoptosis and faulty nuclear remodelling during spermatogenesis. RBM Online.

[B4] Oosterhuis GJ, Mulder AB, Kalsbeek-Batenburg E, Lambalk CB, Schoemaker J, Vermes I (2000). Measuring apoptosis in human spermatozoa: a biological assay for semen quality. Fertil Steril.

[B5] Kasai T, Ogawa K, Mizuno K, Nagai S, Uchida Y, Ohta S, Fujie M, Suzuki K, Hirata S, Hoshi K (2002). Relationship between sperm mitochondrial membrane potential, sperm motility, and fertility potential. Asian J Androl.

[B6] Weng SL, Taylor SL, Morshedi M, Schuffner A, Duran EH, Beebe S, Oehninger S (2002). Caspase activity and apoptotic markers in ejaculated human sperm. Mol Hum Reprod.

[B7] Taylor SL, Weng SL, Fox P, Duran EH, Morshedi MS, Oehninger S, Beebe SJ (2004). Somatic cell apoptosis markers and pathways in human ejaculated sperm: potential utility as indicators of sperm quality. Mol Hum Reprod.

[B8] Seli E, Gardner DK, Schoolcraft WB, Moffatt O, Sakkas D (2004). Extend of nuclear DNA damage in ejaculated spermatozoa impacts on blastocyst development after in vitro fertilization. Fertil Steril.

[B9] Flesch FM, Gadella BM (2000). Dynamics of the mammalian sperm plasma membrane in the process of fertilization. Biochim Biophys Acta.

[B10] Flesch FM, Brouwers JF, Nievelstein PF, Verkleij AJ, van Golde LM, Colenbrander B, Gadella BM (2001). Bicarbonate stimulated phospholipid scrambling induces cholesterol redistribution and enables cholesterol depletion in the sperm plasma membrane. J Cell Sci.

[B11] Gadella BM, Harrison RA (2002). Capacitation induces cyclic adenosine 3',5'-monophosphate-dependent, but apoptosis-unrelated, exposure of aminophospholipids at the apical head plasma membrane of boar sperm cells. Biol Reprod.

[B12] Kotwicka M, Jendraszak M, Warchol JB (2002). Plasma membrane translocation of phosphatidylserine in human spermatozoa. Folia Histochem Cytobiol.

[B13] Lefièvre L, Jha KN, de Lamirande E, Visconti PE, Gagnon C (2002). Activation of protein kinase A during human sperm capacitation and acrosome reaction. J Androl.

[B14] Cross NL (2003). Decrease in order of human sperm lipids during capacitation. Biol Reprod.

[B15] de Vries KJ, Wiedmer T, Sims PJ, Gadella BM (2003). Caspase-independent exposure of aminophospholipids and tyrosine phosphorylation in bicarbonate responsive human sperm cells. Biol Reprod.

[B16] Salicioni AM, Platt MD, Wertheimer EV, Arcelay E, Allaire A, Sosnik J, Visconti PE (2007). Signalling pathways involved in sperm capacitation. Soc Reprod Fertil Suppl.

[B17] Martin SJ, Reutelingsperger CP, McGahon AJ, Rader JA, van Schie RC, LaFace DM, Green DR (1995). Early redistribution of plasma membrane phosphatidylserine is a general feature of apoptosis regardless of initiating stimulus: inhibition by overexpression of Bcl-2 and Abl. J Exp Med.

[B18] Schlegel RA, Williamson P (2001). Phosphatidylserine, a death knell. Cell Death Differ.

[B19] Fadeel B (2004). Plasma membrane alterations during apoptosis: role in corpse clearance. Antioxid Redox Signal.

[B20] Wu Y, Tibrewal N, Birge RB (2006). Phosphatidylserine recognition by phagocytes: a view to a kill. Trends Cell Biol.

[B21] Van Heerde WL, de Groot PG, Reutelingsperger CP (1995). The complexity of the phospholipid binding protein Annexin V. Thromb Haemost.

[B22] Vermes I, Haanen C, Steffens-Nakken H, Reutelingsperger C (1995). A novel assay for apoptosis. Flow cytometric detection of phosphatidylserine expression on early apoptotic cells using fluorescein labelled Annexin V. J Immunol Methods.

[B23] Rodriguez I, Ody C, Araki K, Garcia I, Vassalli P (1997). An early and massive wave of germinal cell apoptosis is required for the development of functional spermatogenesis. EMBO J.

[B24] Tesarik J, Greco E, Cohen-Bacrie P, Mendoza C (1998). Germ cell apoptosis in men with complete and incomplete spermiogenesis failure. Mol Hum Reprod.

[B25] Kawasaki Y, Nakagawa A, Nagaosa K, Shiratsuchi A, Nakanishi Y (2002). Phosphatidylserine binding of class B scavenger receptor type I, a phagocytosis receptor of testicular sertoli cells. J Biol Chem.

[B26] Sakkas D, Seli E, Bizzaro D, Tarozzi N, Manicardi GC (2003). Abnormal spermatozoa in the ejaculate: abortive apoptosis and faulty nuclear remodelling during spermatogenesis. RBM Online.

[B27] Maeda Y, Shiratsuchi A, Namiki M, Nakanishi Y (2002). Inhibition of sperm production in mice by annexin V microinjected into seminiferous tubules: possible etiology of phagocytic clearance of apoptotic spermatogenic cells and male infertility. Cell Death Differ.

[B28] Oehninger S, Morshedi M, Weng SL, Taylor S, Duran H, Beebe S (2003). Presence and significance of somatic cell apoptosis markers in human ejaculated spermatozoa. Reprod Biomed Online.

[B29] Müller K, Pomorski T, Muller P, Herrmann A (1999). Stability of transbilayer phospholipid asymmetry in viable ram sperm cells after cryotreatment. J Cell Sci.

[B30] Marti E, Pérez-Pé R, Colas C, Muino-Blanco T, Cebrian-Pérez JA (2008). Study of apoptosis-related markers in ram spermatozoa. Anim Reprod Sci.

[B31] Pena FJ, Johannisson A, Wallgren M, Rodriguez-Martinez H (2003). Assessment of fresh and frozen-thawed boar semen using an annexin-V assay: a new method of evaluating sperm membrane integrity. Theriogenology.

[B32] Kurz A, Viertel D, Herrmann A, Müller K (2005). Localization of phosphatidylserine in boar sperm cell membranes during capacitation and acrosome reaction. Reproduction.

[B33] Anzar M, He L, Buhr MM, Kroetsch TG, Pauls KP (2002). Sperm apoptosis in fresh and cryopreserved bull semen detected by flow cytometry and its relationship with fertility. Biol Reprod.

[B34] Januskauskas A, Johannisson A, Rodriguez-Martinez H (2003). Subtle membrane changes in cryopreserved bull semen in relation with sperm viability, chromatin structure, and field fertility. Theriogenology.

[B35] Martin G, Sabido O, Durand P, Levy R (2004). Cryopreservation induces an apoptosis-like mechanism in bull sperm. Biol Reprod.

[B36] Glander HJ, Schaller J (1999). Binding of annexin V to plasma membranes of human spermatozoa: a rapid assay for detection of membrane changes after cryostorage. Mol Hum Reprod.

[B37] James PS, Wolfe CA, Mackie A, Ladha S, Prentice A, Jones R (1999). Lipid dynamics in the plasma membrane of fresh and cryopreserved human spermatozoa. Hum Reprod.

[B38] Duru NK, Morshedi M, Schuffner A, Oehninger S (2001). Cryopreservation-thawing of fractionated human spermatozoa is associated with membrane phosphatidylserine externalization and not DNA fragmentation. J Androl.

[B39] Grunewald S, Paasch U, Glander HJ (2001). Enrichment of non-apoptotic human spermatozoa after cryopreservation by immunomagnetic cell sorting. Cell and Tissue Bank.

[B40] Grunewald S, Baumann T, Paasch U, Glander HJ (2006). Capacitation and acrosome reaction in nonapoptotic human spermatozoa. Ann NY Acad Sci.

[B41] Schuffner A, Morshedi M, Oehninger S (2001). Cryopreservation of fractionated, highly motile human spermatozoa: effect on membrane phosphatidylserine externalization and lipid peroxidation. Hum Reprod.

[B42] Paasch U, Sharma RK, Gupta AK, Grunewald S, Mascha EJ, Thomas AJ, Glander HJ, Agarwal A (2004). Cryopreservation and thawing is associated with varying extent of activation of apoptotic machinery in subsets of ejaculated human spermatozoa. Biol Reprod.

[B43] Sion B, Janny L, Boucher D, Grizard G (2004). Annexin V binding to plasma predicts the quality of human cryopreserved spermatozoa. Int J Androl.

[B44] Barroso G, Morshedi M, Oehninger S (2000). Analysis of DNA fragmentation, plasma membrane translocation of phosphatidylserine and oxidative stress in human spermatozoa. Hum Reprod.

[B45] Barroso G, Taylor S, Morshedi M, Manzur F, Gavino F, Oehninger S (2006). Mitochondrial membrane potential integrity and plasma membrane translocation of phosphatidylserine as early apoptotic markers: a comparison of two different sperm populations. Fertil Steril.

[B46] Oosterhuis GJ, Mulder AB, Kalsbeek-Batenburg E, Lambalk CB, Schoemaker J, Vermes I (2000). Measuring apoptosis in human spermatozoa: a biological assay for semen quality. Fertil Steril.

[B47] Ricci G, Perticarari S, Fragonas E, Giolo E, Canova S, Pozzobon C, Guaschino S, Presani G (2002). Apoptosis in human sperm: its correlation with semen quality and the presence of leukocytes. Hum Reprod.

[B48] Schuffner A, Morshedi M, Vaamonde D, Duran EH, Oehninger S (2002). Effect of different incubation conditions on phosphatidylserine externalization and motion parameters of purified fractions on highly motile human spermatozoa. J Androl.

[B49] Shen HM, Dai J, Chia SE, Lim A, Ong CN (2002). Detection of apoptotic alterations in sperm in subfertile patients and their correlations with sperm quality. Hum Reprod.

[B50] Paasch U, Grunewald S, Fitzl G, Glander HJ (2003). Deterioration of plasma membrane is associated with activated caspases in human spermatozoa. J Androl.

[B51] Paasch U, Grunewald S, Wuendrich K, Jope T, Glander HJ (2005). Immunomagnetic removal of cryo-damage human spermatozoa. Asian J of Androl.

[B52] Lachaud C, Tesarik J, Canadas ML, Mendoza C (2004). Apoptosis and necrosis in human ejaculated spermatozoa. Hum Reprod.

[B53] Moustafa MH, Sharma RK, Thornton J, Mascha E, Abdel-Hafez MA, Thomas AJ, Agarwal A (2004). Relationship between ROS production, apoptosis and DNA denaturation in spermatozoa from patients examined for infertility. Hum Reprod.

[B54] Martin G, Sabido O, Durand P, Levy R (2005). Phosphatidylserine externalization in human sperm induced by calcium ionophore A23187: relationship with apoptosis, membrane scrambling and the acrosome reaction. Human Reprod.

[B55] Chen Z, Hauser R, Trbovich AM, Shifren JL, Dorer DJ, Godfrey-Bailey L, Singh NP (2006). The relationship between human semen characteristics and sperm apoptosis: a pilot study. J Androl.

[B56] Zhang HB, Chen ZJ, Ma CY, Lu SM, Wang L, Li X (2008). Early apoptotic changes in human spermatozoa and their relationships with conventional semen parameters and sperm DNA fragmentation. Asian J Androl.

[B57] Muratori M, Porazzi I, Luconi M, Marchiani S, Forti G, Baldi E (2004). AnnexinV binding and merocyanine staining fail to detect human sperm capacitation. J Androl.

[B58] Sakkas D, Moffatt O, Manicardi GC, Mariethoz E, Tarozzi N, Bizzaro D (2002). Nature of DNA damage in ejaculated human spermatozoa and the possible involvement of apoptosis. Biol Reprod.

[B59] Cayli S, Sakkas D, Vigue L, Demir R, Huszar G (2004). Cellular maturity and apoptosis in human sperm: creatine kinase, caspase-3 and Bcl-XL levels in mature and diminished maturity sperm. Mol Hum Reprod.

[B60] Aitken RJ, Buckingham D, West K, Wu FC, Zikopoulos K, Richardson DW (1992). Differential contribution of leukocytes and spermatozoa to the generation of reactive oxygen species in the ejaculates of oligozoospermic patients and fertile donors. J Reprod Fertil.

[B61] Ochsendorf FR (1999). Infections in the male genital tract and reactive oxygen species. Hum Reprod Update.

[B62] Lessig J, Spalteholz H, Reibetanz U, Salavei P, Fischlechner M, Glander HJ, Arnhold J (2007). Myeloperoxidase binds to non-vital spermatozoa on phosphatidylserine epitopes. Apoptosis.

[B63] Villegas J, Schulz M, Soto L, Sanchez R (2005). Bacteria induce expression of apoptosis in human spermatozoa. Apoptosis.

[B64] Glander HJ, Schiller J, Süss R, Paasch U, Grunewald S, Arnhold J (2002). Deterioration of spermatozoal plasma membrane is associated with an increase of sperm lyso-phosphatidylcholines. Andrologia.

[B65] Said TM, Grunewald S, Paasch U, Glander HJ, Baumann T, Kriegel C, Li L, Agarwal A (2005). Advantage of combining magnetic cell separation with sperm preparation techniques. RBM Online.

[B66] Said TM, Grunewald S, Paasch U, Rasch M, Agarwal A, Glander HJ (2005). Effects of magnetic-activated cell sorting on sperm motility and cryosurvival rates. Fertil Steril.

[B67] Said TM, Aziz N, Sharma RK, Lewis-Jones I, Thomas AJ, Agarwal A (2005). Novel association between sperm deformity index and oxidative stress-induced DNA damage in infertile male patients. Asian J Androl.

[B68] Said TM, Agarwal A, Grunewald S, Rasch M, Glander HJ, Paasch U (2006). Evaluation of sperm recovery following annexin V magnetic-activated cell sorting separation. RBM Online.

[B69] Said TM, Agarwal A, Grunewald S, Rasch M, Baumann T, Kriegel C, Li L, Glander HJ, Thomas AJ, Paasch U (2006). Selection of nonapoptotic spermatozoa as a new tool for enhancing assisted reproduction outcomes: an in vitro model. Biol Reprod.

[B70] Said TM, Agarwal A, Zborowski M, Grunewald S, Glander HJ, Paasch U (2008). Utility of magnetic cell separation as a molecular sperm preparation technique. J Androl.

[B71] Miltenyi S, Muller W, Weichel W, Radbruch A (1990). High gradient magnetic cell separation with MACS. Cytometry.

[B72] Auger J, Leonce S, Jouannet P, Ronot X (1993). Flow cytometric sorting of living, highly motile spermatozoa based on evaluation of their mitochondrial activity. J Histochem Cytochem.

[B73] Marchetti C, Marchetti P (2005). Place des marqueurs de l'apoptose dans l'exploration de l'infertilité masculine. Gynecol Obstet Fertil.

[B74] Henkel RR, Schill WB (2003). Sperm preparation for ART. Reprod Biol Endocrinol.

[B75] Tesarik J (1989). Appropriate timing of the acrosome reaction is a major requirement for the fertilizing spermatozoon. Hum Reprod.

[B76] Matthijs A, Harkema W, Engel B, Woelders H (2000). In vitro phagocytosis of boar spermatozoa by neutrophils from peripheral blood of sows. J Reprod Fertil.

[B77] Henkel R, Hajimohammad M, Stalf T, Hoogendijk C, Mehnert C, Menkveld R, Gips H, Schill WB, Kruger TF (2004). Influence of deoxyribonucleic acid damage on fertilization and pregnancy. Fertil Steril.

[B78] Host E, Lindenberg S, Smidt-Jensen S (2000). The role of DNA strand breaks in human spermatozoa used for IVF and ICSI. Acta Obstet Gynecol Scand.

[B79] Paasch U, Grunewald S, Glander HJ (2007). Sperm selection in assisted reproductive techniques. Soc Reprod Fertil Suppl.

[B80] Gallon F, Marchetti C, Jouy N, Marchetti P (2006). The functionality of mitochondria differentiates human spermatozoa with high and low fertilizing capability. Fertil Steril.

[B81] Grunewald S, Paasch U, Said TM, Rasch M, Agarwal A, Glander HJ (2006). Magnetic-activated cell sorting before cryopreservation preserves mitochondrial integrity in human spermatozoa. Cell Tissue Bank.

[B82] Aitken RJ, Buckingham D, West K, Wu FC, Zikopoulos K, Richardson DW (1992). Differential contribution of leukocytes and spermatozoa to the generation of reactive oxygen species in the ejaculates of oligozoospermic patients and fertile donors. J Reprod Fertil.

[B83] Sharma RK, Pasqualotto FF, Nelson DR, Thomas AJ, Agarwal A (1999). The reactive oxygen species-total antioxidant capacity score is a new measure of oxidative stress to predict male infertility. Hum Reprod.

[B84] Saleh RA, Agarwal A, Kandirali E, Sharma RK, Thomas AJ, Nada EA, Evenson DP, Alvarez JG (2002). Leukocytospermia is associated with increased reactive oxygen species production by human spermatozoa. Fertil Steril.

[B85] Wang X, Sharma RK, Sikka SC, Thomas AJ, Falcone T, Agarwal A (2003). Oxidative stress is associated with increased apoptosis leading to spermatozoa DNA damage in patients with male factor infertility. Fertil Steril.

[B86] Nallella KP, Sharma RK, Allamanene SSR, Agarwal A (2005). Identification of male factor infertility using a novel semen quality score and reactive oxygen species levels. Clinics.

[B87] Athayde KS, Cocuzza M, Agarwal A, Krajcir N, Lucon AM, Srougi M, Hallak J (2007). Development of normal reference values for seminal reactive oxygen species and their correlation with leukocytes and semen parameters in a fertile population. J Androl.

[B88] Deepinder F, Chowdary HT, Agarwal A (2007). Role of metabolic analysis of biomarkers in the management of male infertility. Expert Rev Mol Diagn.

[B89] Moskovtsev SI, Willis J, White J, Mullen JB (2007). Leukocytospermia: relationship to sperm deoxyribonucleic and integrity in patients evaluated for male factor infertility. Fertil Steril.

[B90] Agarwal A, Makler K, Sharma R (2008). Clinical relevance of oxidative stress in male factor infertility: an update. Am J Reprod Immunol.

[B91] Grunewald S, Said TM, Paasch U, Glander HJ, Agarwal A (2008). Relationship between sperm apoptosis signalling and oocyte penetration capacity. Int J Androl.

[B92] Aziz N, Said T, Paasch U, Agarwal A (2007). The relationship between human sperm apoptosis, morphology and the sperm deformity index. Hum Reprod.

[B93] Varum S, Bento C, Sousa APM, Gomes-Santos CSS, Henriques NP, Almeida-Santos T, Teodosio C, Paiva A, Ramalho-Santos J (2007). Characterization of human sperm populations using conventional parameters, surface ubiquitination, and apoptotic markers. Fertil Steril.

[B94] Grunewald S, Miska W, Miska G, Rasch M, Reinhardt M, Glander HJ, Paasch U (2007). Molecular glass wool filtration as a new tool for sperm preparation. Hum Reprod.

